# IRIDEX MicroPulse P3: innovative cyclophotocoagulation

**DOI:** 10.22336/rjo.2017.20

**Published:** 2017

**Authors:** M. Monica Gavris, Ioana Olteanu, Erzsebet Kantor, Radu Mateescu, Roxana Belicioiu

**Affiliations:** * Laser Optisan Clinic, Cluj-Napoca, Romania

**Keywords:** IRIDEX, MicroPulse P3, IOP, glaucoma, cyclophotocoagulation, laser

## Abstract

**Purpose:** To present the new IRIDEX MicroPulse P3 (MP3) technology in patients with refractory glaucoma and our preliminary results at 1 week and 1 month postoperatively.

**Methods:** IRIDEX MP3 laser cyclophotocoagulation was performed in 7 eyes of 7 patients under retrobulbar anaesthesia with lidocaine 2% in the operating room. Each eye received two treatments of 80-90s over the superior and inferior hemisphere, avoiding the temporal- and nasal-most clock hours. 810nm IRIDEX MP3 was set to 31,3% duty cycle (0,5ms treatment pulse followed by 1,1 ms of rest). Postoperative topical steroids were prescribed for 1 week.

**Results:** Mean IOP decrease at 1 week was 60,3% and 33,4% at 1 month, with a mean topical hypotensive treatment reduction of 0,71 therapeutic agents. The procedure was safe in all cases and effective in 71% of the patients. Neovascular glaucoma patients registered high IOP levels 1 month postoperatively in spite of medical and MP3 laser treatment. BCVA remained unchanged after undertaking the laser procedure. No significant inflammation, discomfort, or pain was reported. There were no complications such as hypotony, phthisis bulbi, and macular edema.

**Conclusions:** IRIDEX MP3 represents an innovation in cyclophotocoagulation. It is non-destructive, repeatable, non-invasive, with a high safety profile. A mean IOP decrease of 33,4% was registered at 1 month. Patient comfort and recovery are favorable. Long-term results will prove its efficacy in the future.

## Introduction

Traditional cyclophotocoagulation was introduced as a treatment method to reduce IOP by decreasing the production of aqueous humour via the destruction of the ciliary epithelium. 

The method was initially applied to patients with recalcitrant glaucoma and few treatment options, but gained popularity in recent years and was found more effective than the medical therapy of lowering the IOP, but determined a series of complications: hypotony (up to 10% of the cases), phthisis bulbi, and macular oedema [**1**]. Therefore, the development of a new IOP lowering device with a greater safety profile was necessary. 

**Fig. 1:**
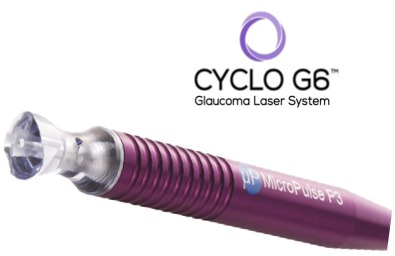
IRIDEX MicroPulse P3 treatment probe

The Micropulse P3 glaucoma device powered by Cyclo G6 Glaucoma Laser System revolutionized cyclophotocoagulation. The new technology takes a continuous wave of laser and breaks it into a series of repetitive pulses separated by pauses that prevent thermal build-up in the tissue [**2**]. This is the first non-incisional, non-invasive laser for the treatment of glaucoma that has a favorable profile and a series of unique features. The procedure can be repeated as much as needed to treat the patient regardless of the previous glaucoma therapy. Particularly good candidates for this therapy method are patients who live alone, for whom surgery would be difficult and inconvenient and patients with compliance difficulties, unable to follow a long-term treatment scheme [**3**] (**Fig. 1**). 

**Purpose**

The aim of our study was to present the new IRIDEX MicroPulse P3 (MP3) technology in patients with refractory glaucoma and our preliminary results at 1 week and 1 month postoperatively.

## Patients and Methods

**Patients**

A prospective case series included 7 eyes of 7 patients with narrow-angle glaucoma [**2**], open-angle glaucoma [**2**], inflammatory glaucoma [**1**] and neovascular glaucoma [**2**], all female patients aged between 21 and 73 years, with a mean age of 60,85 years, treated in November 2016, at Laser Optisan Clinic, Cluj-Napoca.

The following baseline data were collected prior to the treatment: age, sex, ocular history, glaucoma diagnosis-nonresponsive to maximal medical therapy with or without previous surgical intervention, best-corrected Snellen visual acuity (BCVA), glaucoma medications, slit-lamp examination findings of the anterior and posterior pole. All patients signed an informed consent. IRIDEX MP3 laser cyclophotocoagulation was performed in all cases without complications during or after the procedure.

**Method**

**Fig. 2:**
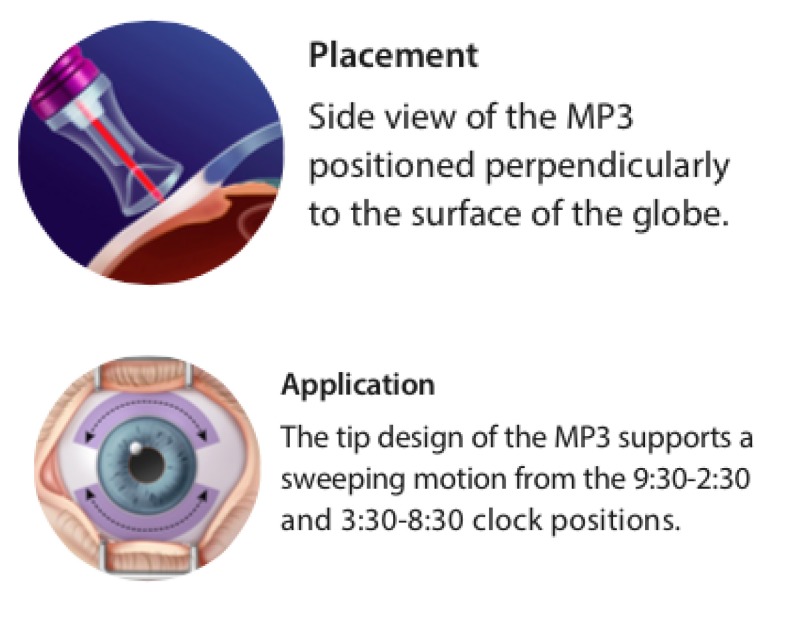
IRIDEX MicroPulse P3 treatment method

The procedure was performed in the operating room, following retrobulbar anaesthesia with lidocaine 2%, each eye received two treatments of 80-90s over the superior and inferior hemisphere avoiding the temporal- and nasal-most clock hours, by moving the probe over 6 clock hours at every 10 seconds, perpendicular and posterior to the limbus [**2**] (**Fig. 2**). 

**Fig. 3:**
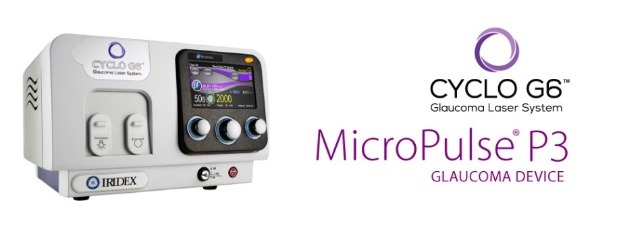
MicroPulse P3 device

The treatment was carried out with the 810nm IRIDEX MP3 laser set to a 31,3% duty cycle (0,5ms treatment pulse followed by 1,1ms of rest) (**Fig. 3**).

Glaucoma treatment was not discontinued and the patients were examined 1 week and 1 month postoperatively. The surgeon was able to start reduce the hypotensive medication depending on how quickly the pressure fell to the value of 10mmHg or below. 

## Results

**Fig. 4:**
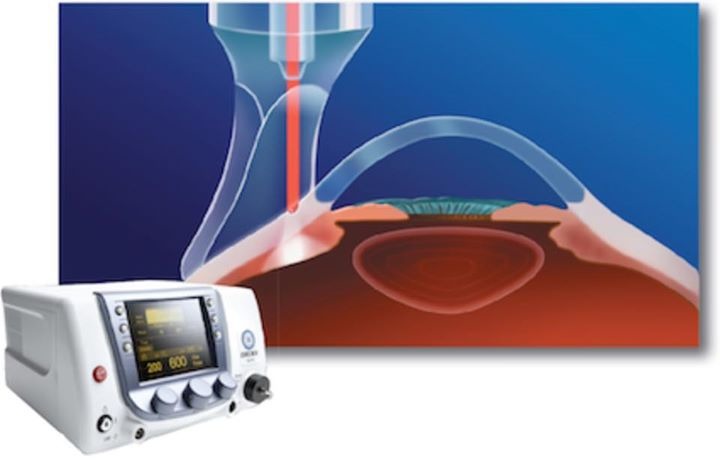
MP3 laser delivery

Patients were examined at 1 week and 1 month postoperatively. The recovery period was favorable and patients did not experience any significant discomfort or pain. MP3 does not destroy the eye’s ciliary body or cause inflammation. Because it is a non-incisional procedure, in which the system’s probe is placed directly on the sclera [**4**], the risk of bleeding and infection is eliminated (**Fig. 4**).

A mean IOP decrease of 60,3% was observed at 1 week postoperatively (34,7% to 71,9% from baseline) and 33,4% 1 month after the procedure (0% to 60,8% from baseline). In 3 cases, medication was reduced by 1 therapeutic agent, in 1 case by 2 and in 3 cases medical treatment remained unchanged after 1 month, resulting in a mean reduction of topical hypotensive medication of 0,71 agents. Best corrected Snellen VA was stable before and after treatment, ranging from wlp to 0,8. 

The MP3 laser treatment was considered successful in 71% of the cases. Unsatisfactory results were obtained in 2 cases of neovascular glaucoma, a secondary glaucoma generally associated with a poor prognosis. If clinical hypotensive treatment is not sufficient, a more complex approach is required: panretinal photocoagulation, intravitreal anti-VEGF therapies, and surgical procedures for intraocular pressure control [**5**].

All patients were re-examined at 3, 6, 12, and 18 months postoperatively and retreatment could be performed within the 18 months follow-up period (**Table 1**).

**Table 1:** 18 months follow-up period of the patients

	Preop IOP	Preop treatment	DT (S)	1 week postop PIO	1 week postop PIO change	Treatment after 1 month	1 month postop PIO	1 month postop PIO change
Case #1 PNAG 59 years old	26	5	80	12	53,8%	3	20	23%
Case #2 PNAG 71 years old	33	4	80	12	63,6%	3	20	39,3%
Case #3 POAG 73 years old	23	5	80	15	34,7%	4	14	39,1%
Case #4 POAG 61 years old	40	5	90	14	65%	4	21	47,5%
Case #5 SIG	46	3	90	13,7	70,2%	3	18	60,8%
Case #6 SNG	57	2	90	16	71,9%	2	43	24,5%
Case #7 SNG	27	2	90	10	62,9%	2	27	0%
• All patients received a retrobulbar injection of 2% lidocaine								
• All cases were treated with a range of 80-90 seconds per hemisphere and 2000 mW								

## Discussions 

Cyclophotocoagulation with the IRIDEX MicroPulse P3 device represents a new tissue-sparing technology used in simple and complex glaucoma cases. While standard cyclophotocoagulation (CPC) involves ciliary body destruction by targeting the ciliary epithelium and stroma resulting in a reduction in aqueous secretion and IOP, MP3 administers a series of repetitive, short pulses of laser energy separated by rest periods, unlike conventional CPC, which delivers continuous, high intensity energy to the ciliary body [**6**]. 

**Fig. 5:**
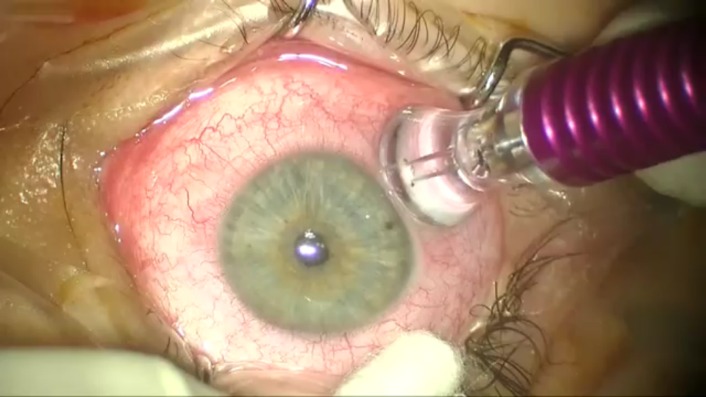
IRIDEX MP3 procedure

The procedure is non-invasive, easily undertaken, and well tolerated, with transscleral application, eliminating bleeding and postoperative infection risks. Postoperative inflammation and pain are insignificant and hospital admission is not required (**Fig. 5**).

1 week postoperatively, a mean IOP decrease of 60,3% was observed, under unchanged glaucoma medication. 1 month after the laser procedure, the average IOP decreased by 33,4% from baseline, combined with a mean reduction of 0,71 therapeutic agents in ocular hypotensive medications.

The treatment was delivered with an excellent safety profile. No cases of hypotony, phthisis bulbi, or macular edema were observed [**7**].

IRIDEX MP3 cyclophotocoagulation was successful in 71% of the cases. Unsatisfactory IOP values were observed at 1 month in neovascular glaucoma patients: the first case presented a mean IOP decrease of 24,5% but remained outside the normal range and the second case presented no IOP decrease from baseline at 1 month. These results showed the need of a more complex therapeutic approach in such cases, where the IOP lowering treatment was not enough.

Patients were re-examined at 3, 6, 12 and 18 months postoperatively. If IOP reached values of 10mmHg or below, glaucoma medication could be reduced. Retreatments could be performed at 6-8 weeks after the first treatment, within the 18 months follow-up period, if required.

## Conclusions

1. IRIDEX MicroPulse P3 represents an innovation in cyclophotocoagulation. MP3 programming takes a continuous wave and breaks it into a series of short, repetitive pulses separated by pauses in order to prevent thermal build-up and damage.

2. MP3 therapy is a non-destructive, repeatable, non-invasive procedure, with a high safety profile. 

3. A mean IOP decrease of 33,4% was registered 1 month postoperatively. Patient comfort and recovery were favorable. There was less inflammation, no bleeding and no infection risk associated with the procedure.

4. Further studies and results will prove its long-term efficacy in the future.
